# Shedding of bevacizumab in tumour cells-derived extracellular vesicles as a new therapeutic escape mechanism in glioblastoma

**DOI:** 10.1186/s12943-018-0878-x

**Published:** 2018-08-31

**Authors:** Thomas Simon, Sotiria Pinioti, Pascale Schellenberger, Vinothini Rajeeve, Franz Wendler, Pedro R. Cutillas, Alice King, Justin Stebbing, Georgios Giamas

**Affiliations:** 1 Department of Biochemistry and Biomedicine, University of Sussex, School of Life Sciences, Brighton, BN1 9QG UK; 2 Present address: Laboratory of Tumor Inflammation and Angiogenesis, Center for Cancer Biology (CCB), VIB, Leuven, Belgium; 3 Cell Signalling & Proteomics Group, Centre for HaematoOncology, Barts Cancer Institute, Queen Mary University of London, London, UK; 4 Department of Surgery and Cancer, Hammersmith Hospital Campus, Imperial College London, Division of Cancer, Du Cane Road, London, W12 0NN UK

**Keywords:** Bevacizumab, Extracellular vesicles, Glioblastoma, Resistance

## Abstract

Glioblastoma (GBM) is the most aggressive type of primary brain tumours. Anti-angiogenic therapies (AAT), such as bevacizumab, have been developed to target the tumour blood supply. However, GBM presents mechanisms of escape from AAT activity, including a speculated direct effect of AAT on GBM cells. Furthermore, bevacizumab can alter the intercellular communication of GBM cells with their direct microenvironment. Extracellular vesicles (EVs) have been recently described as main acts in the GBM microenvironment, allowing tumour and stromal cells to exchange genetic and proteomic material. Herein, we examined and described the alterations in the EVs produced by GBM cells following bevacizumab treatment. Interestingly, bevacizumab that is able to neutralise GBM cells-derived VEGF-A, was found to be directly captured by GBM cells and eventually sorted at the surface of the respective EVs. We also identified early endosomes as potential pathways involved in the bevacizumab internalisation by GBM cells. Via MS analysis, we observed that treatment with bevacizumab induces changes in the EVs proteomic content, which are associated with tumour progression and therapeutic resistance. Accordingly, inhibition of EVs production by GBM cells improved the anti-tumour effect of bevacizumab. Together, this data suggests of a potential new mechanism of GBM escape from bevacizumab activity.

**Electronic supplementary material:**

The online version of this article (10.1186/s12943-018-0878-x) contains supplementary material, which is available to authorized users.

GBM is amongst the most aggressive types of brain tumours for which current treatments are of limited benefit [1]. During the past decades, AAT have provided a rationale for targeting and blocking the tumour blood supply. Unfortunately, the effects of AAT/bevacizumab, a monoclonal humanised antibody neutralising Vascular Endothelial Growth Factor-A (VEGF-A), on tumour growth are short-term and GBM patients ultimately relapse. Interestingly, since the expression of some pro-angiogenic factors and their receptors (i.e. VEGF-A/VEGF-R) has been described in tumour cells, it appears that AAT/bevacizumab also acts directly on GBM cells [2] that might eventually lead to therapy resistance and relapse [1]. Recently, we identified a direct effect of bevacizumab on GBM cells and demonstrated its ability to stimulate tumour cells’ invasion in hyaluronic acid (HA) hydrogels and activate key survival signalling pathways. The intrinsic reluctance of cancer cells to AAT could also be linked to their ability of disposing the drugs [3]. Indeed, it has been observed that cetuximab, an EGF-R monoclonal IgG1 antibody, is associated with extracellular vesicles (EVs) derived from treated cancer cells suggesting that such processes could be implicated in tumour limited response to therapy [4]. During the last years, EVs involvement in tumour development and metastasis has been thoroughly considered [5]. Therefore, herein we focused on the effects of bevacizumab on the production of GBM cells-derived EVs.

## Results/discussion

### Bevacizumab affects the EVs proteomic content derived from GBM cells

**Fig. 1:**
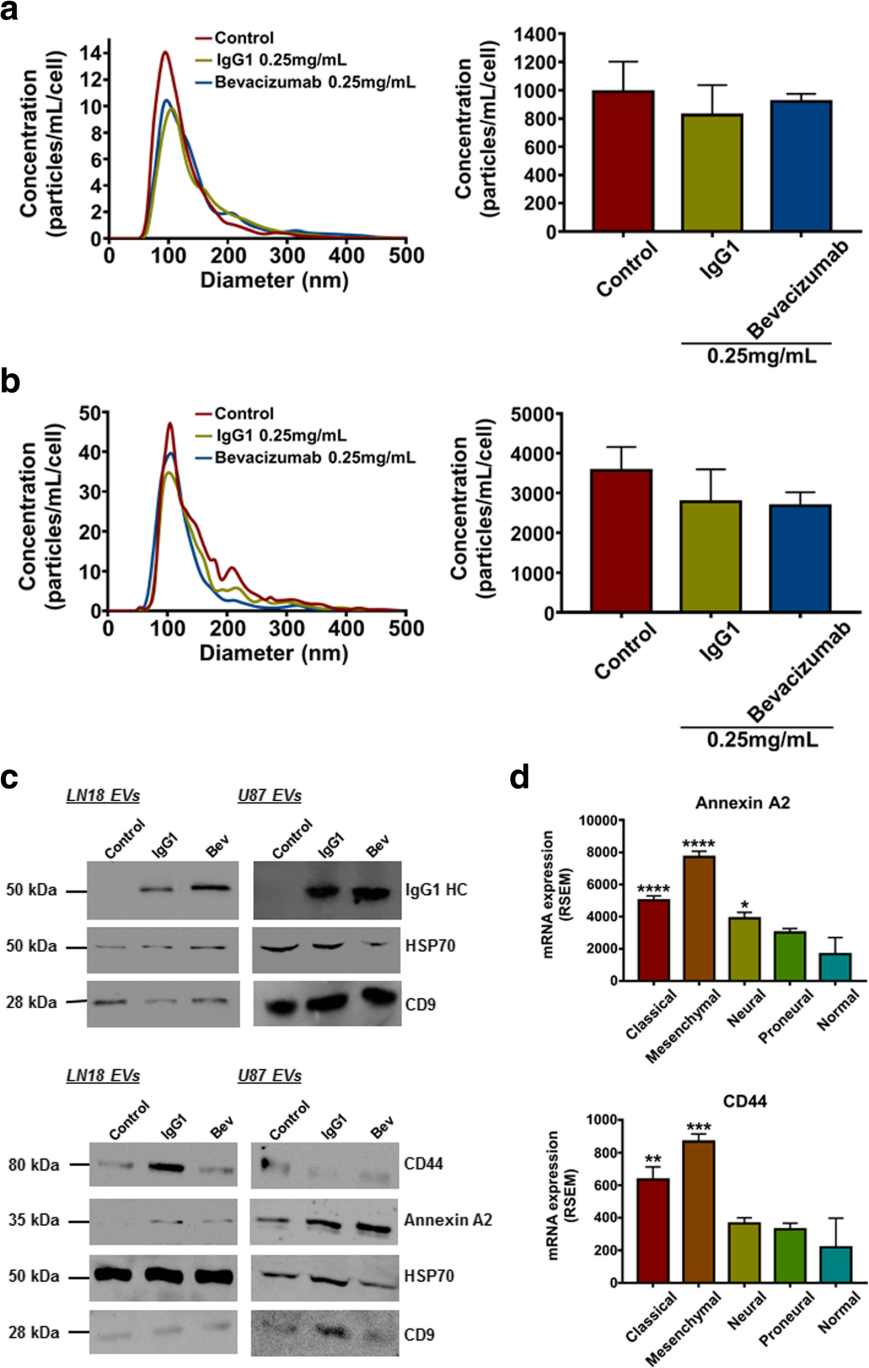
IgG1/Bevacizumab antibody can affect LN18 and U87 GBM cells-derived EVs concentration and their proteomic content. NTA of **a** LN18 or **b** U87 GBM cells-derived EVs following treatment with bevacizumab (0.25 mg/mL). LN18 or U87 GBM cells were treated for 24 h with 0.25 mg/mL IgG1 or bevacizumab. Then cells were washed two times with sterile PBS and incubated additional 24 h in serum free conditions without treatment. CM was then collected, EVs were isolated and re-suspended in 100 μL filtered sterile PBS. EVs suspension was 1/5 diluted and infused to a Nanosight© NS300 instrument. 5 captures of 60s each were recorded. Particles concentration (particles/mL) and size (nm) were measured. Particles concentration was normalised to the number of cells after treatment (particles/mL/cell). The mean ± SEM of 3 independent experiments is shown. **c** Western blotting validation of the human IgG, Annexin A2 and CD44 in EVs derived from LN18 and U87 GBM cells. **d** Gene expression distribution of Annexin A2 and CD44 among the different GBM subtypes has been obtained from TCGA. The mean ± SEM is shown (*p < 0.05, **p < 0.01, ***p < 0.001,****p < 0.0001; ANOVA, compare to ‘normal’)

Since VEGF-A represents the main target of bevacizumab and in order to determine the best model for our study, we examined the expression of different components of the VEGF-A signalling in three different GBM cell lines (see Additional file 1: for Materials and Methods). As LN18 and U87 secreted the highest amounts of VEGF-A, we decided to focus on the effects of bevacizumab on these cell lines (Additional file 2: Figure S1a, S1b). Although bevacizumab neutralised VEGF-A secreted by LN18 and U87 (Additional file 2: Figure S1c), cell viability and proliferation appeared to be marginally affected with clinically relevant doses (~ 0.25 mg/mL), while the only statistically significant decrease on GBM viability (~ 10%) and proliferation (~ 30%) was observed with high doses (Additional file 2: Figure S1d, S1e). Moreover, nanoparticles tracking analysis (NTA) showed no significant change in the concentration of LN18 or U87 cells-derived EVs (~ 1000 and ~ 3000 particles/mL/cell, respectively) in response to bevacizumab (Fig. 1a, b), while MS analysis showed that treatment with either bevacizumab or control IgG1 could modify the proteomic cargo of EVs derived from GBM cells (Additional file 3: Figure S2 and Additional file 4: Tables S1 and S2). Interestingly, the fact that even ‘unspecific’ IgG1 altered the EVs proteomic cargo, suggests that GBM cells respond to the immunotherapy. Furthermore, immunoglobulins peptides could be noticed in EVs derived from both IgG1- and bevacizumab-treated LN18 and U87, suggesting that the antibody used associates somehow with EVs in a non-VEGF-A specific way. Moreover, Annexin A2 expression, a well-described angiogenesis and tumour progression promoter in gliomas and breast cancer, increased in both LN18 and U87 cells-derived EVs following bevacizumab treatment (Fig. 1c). Annexin A2 has also been described as a new potential marker for GBM aggressiveness and patients’ survival [6]. We also observed a decrease in CD44 expression in U87 cells-derived EVs following bevacizumab treatment. Yet, a decrease in CD44 along with an increase in Annexin A2 might suggest a switch in the EVs sub-populations upon bevacizumab treatment [7]. Finally, respective patients’ gene expression in the different GBM subtypes has been obtained from The Cancer Genome Atlas (TCGA). As illustrated in Fig. 1d, Annexin A2 and CD44 are mostly over-expressed in the classical and mesenchymal subtypes. Overall, further investigation is required to decipher whether EVs derived from bevacizumab treated-GBM cells promote tumour aggressiveness through their protein content.

### Bevacizumab is internalised by GBM cells

**Fig. 2:**
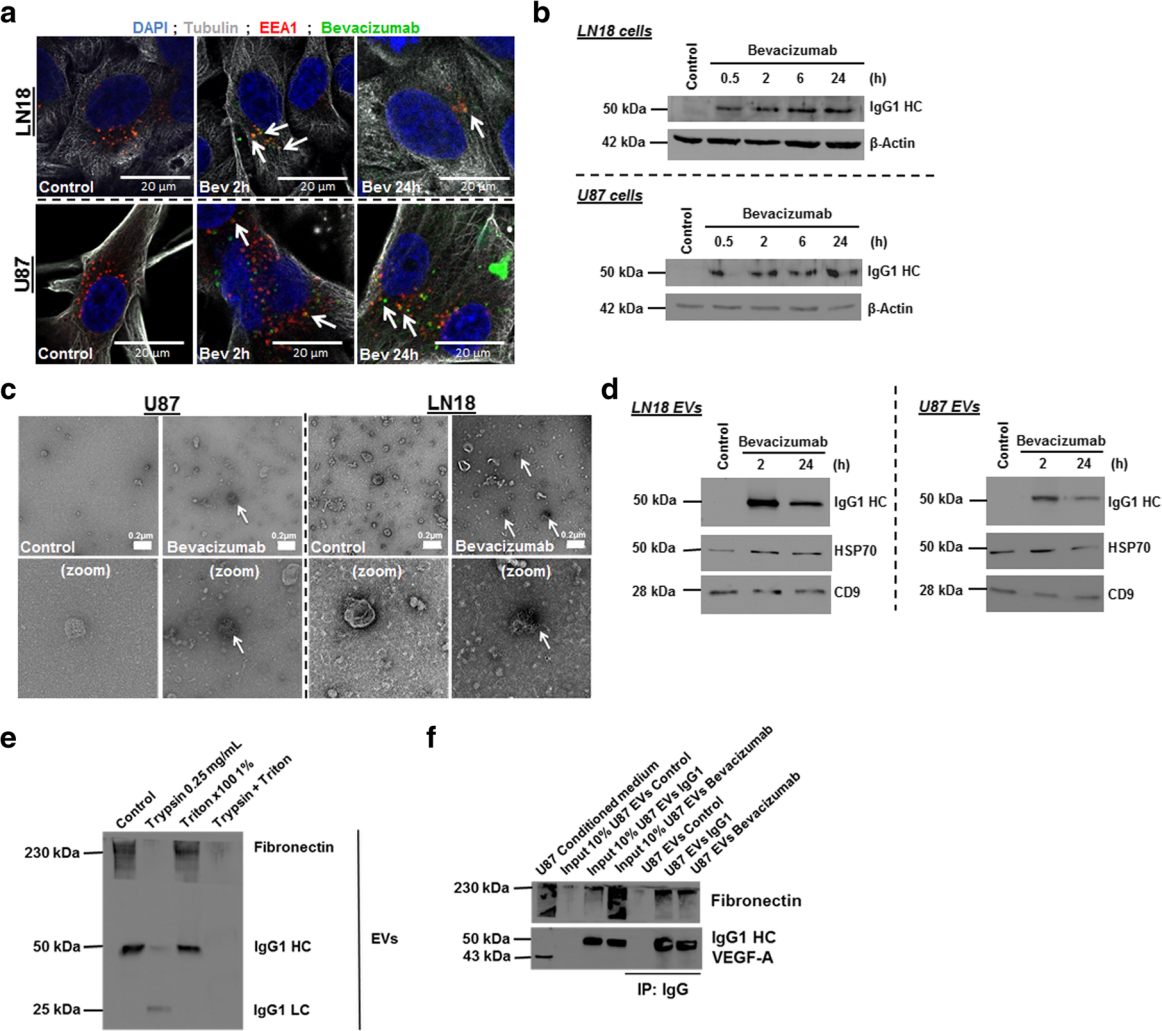
Bevacizumab is internalised by GBM cells and is detectable at the surface of GBM cells-derived EVs following treatment. **a** Immunofluorescence detection of bevacizumab and EEA1 in LN18 and U87 GBM cells. GBM cells were allowed to grow on cover slips and then treated with 0.25 mg/mL bevacizumab for 2 h and 24 h. Cells were fixed with 4% PFA and then incubated with antibodies against α-tubulin, EEA1 and human IgG1. Pictures were taken at × 120 magnification. Representative pictures are shown. **b** Western blotting detection of bevacizumab in LN18 and U87 GBM cells. GBM cells were treated for different times (30 min, 2 h, 6 h and 24 h) with 0.25 mg/mL bevacizumab. Cells were then washed two times with sterile PBS, collected and lysed with RIPA buffer for proteins extraction. β-actin and bevacizumab (IgG1) expression was analyzed by western blotting. Representative pictures are shown. **c** TEM detection of bevacizumab in LN18 and U87 GBM cells-derived EVs. U87 GBM cells were treated for 24 h with 0.25 mg/mL bevacizumab. Then cells were washed two times with sterile PBS and incubated additional 24 h in serum free conditions without treatment. CM was then collected and EVs were isolated. Immuno-gold labeling was then performed against human IgG in the EVs fractions. Pictures were taken at × 20,000 magnification. Representative pictures are shown. **d** Western blotting detection of bevacizumab in LN18 and U87 GBM cells-derived EVs. GBM cells were treated for 2 h and 24 h with 0.25 mg/mL bevacizumab. CM was collected after treatment. Cells were washed twice with sterile PBS and incubate for additional 24 h in serum free condition before CM was collected again and EVs isolated. CD9, HSP70 and bevacizumab (IgG1) expression was analysed by western blotting. A representative picture is shown. **e** Western blotting detection of fibronectin (positive control previously described to be present at the surface of cancer cells EVs) and IgG1 antibody in LN18 GBM cells-derived EVs. Western blotting detection of bevacizumab in LN18 GBM cells-derived EVs. GBM cells were treated for 24 h with 0.25 mg/mL bevacizumab. Then cells were washed two times with sterile PBS and incubated additional 24 h in serum free conditions without treatment. CM was then collected and EVs were isolated. EVs suspension was then diluted in a 2.5 mg/mL trypsin solution or 1% triton X100 or a trypsin/triton combination. Fibronectin and bevacizumab (IgG1) expression was analysed by western blotting. A representative picture is shown. **f** Western blotting detection of IgG1/bevacizumab antibody, fibronectin and VEGF-A in U87 GBM cells-derived EVs. U87 GBM cells were treated for 24 h with 0.25 mg/mL IgG1 or bevacizumab. Cells were washed twice with sterile PBS and incubate for additional 24 h in serum free condition before CM was collected. Then EVs were isolated from CM. IgG1/bevacizumab antibody was precipitated using an immunoprecipitation matrix. Protein extraction was performed on EVs using RIPA buffer. IgG1, fibronectin and VEGF-A expression was analysed by western blotting. A representative picture is shown. IgG1 HC = IgG1 Heavy chains

We then assessed the presence of bevacizumab in GBM cells. As previously observed [8], we showed a partial co-localisation of bevacizumab with EEA1 (and Rab5 in U87) in GBM cells, suggesting that the antibody uptake might involve receptor/ligand dependent endocytosis (Fig. 2a**,** Additional file 5: Figure S3a). Western blotting revealed a time-dependent increase of bevacizumab in treated-GBM cells, suggesting a gradual incorporation of bevacizumab over time (Fig. 2b). This mechanism appears to be quite rapid as suggested by the detection of bevacizumab in EVs following 2 h treatment, which could happen via fast recycling endosomes as recently proposed [9].

### Bevacizumab is detectable at the surface of GBM cells-derived EVs

We hypothesised that IgG1 antibodies could end up at the surface of EVs derived from treated cells. When compared to control EVs, TEM revealed bevacizumab as aggregates on some of the EVs from treated cells, suggesting that the antibody is present at the surface of the vesicles (Fig. 2c). Accordingly, bevacizumab could be observed in the EVs produced by the respective cells following treatment (Fig. 2d). Moreover, trypsin digestion could affect IgG antibodies in EVs, suggesting that bevacizumab is predominantly bound to the surface of EVs and is not internalised in them. (Fig. 2e and Additional file 5: Figure S3b).

Finally, co-immunoprecipitation experiments revealed that EVs-associated bevacizumab could not bind to VEGF-A. As previously suggested [10], we believe that GBM cells derived-EVs trapping of bevacizumab reduces the available number of antibody molecules reaching their specific targets, thus decreasing its efficacy. Interestingly, fibronectin was detected in both IgG1 and bevacizumab-bound solubilised EVs (Fig. 2f). Accordingly, Deissler et al. suggest that endothelial cells could uptake IgG1 antibodies through binding to fibronectin/integrin complexes [8]. Therefore, it is likely that IgG1 antibodies (bevacizumab) get trapped at the cell surface through binding with cell/ECM interaction components (i.e. fibronectin) [8]. Consequently, bevacizumab shedding at the surface of GBM-derived EVs appears as a possible way for cancer cells to shield themselves against treatment.

### Inhibition of EVs production increases the effects of bevacizumab on GBM cells’ viability

**Fig. 3:**
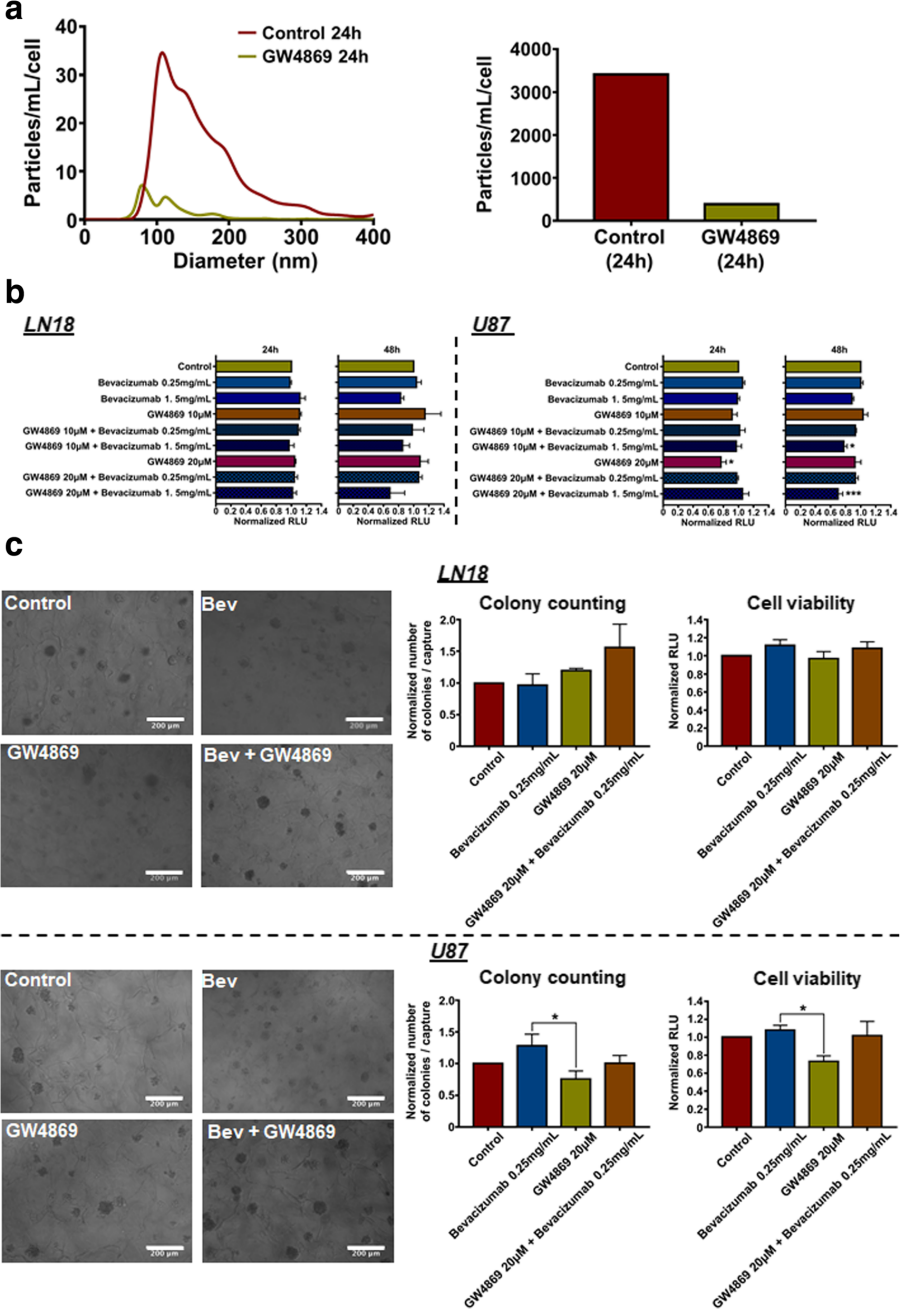
Inhibition of EVs production increases effects of bevacizumab on U87 GBM cells' viability. **a** NTA of LN18 GBM cells-derived EVs following treatment with GW4869 (20 μM). LN18 GBM cells were treated for 24 h with 20 μM GW4869 or DMSO. CM was then collected, EVs were isolated and re-suspended in 100 μL filtered sterile PBS. EVs suspension was 1/5 diluted and infused to a Nanosight© NS300 instrument. 5 captures of 60s each were recorded. Particles concentration (particles/mL) and size (nm) were measured. Particles concentration was normalised to the number of cells after treatment (particles/mL/cell). **b** LN18 and U87 GBM cells' viability assay in response to bevacizumab combined with GW4869. Cells were seeded in a 96well plate and allowed to grow for 24 h. Cells were then treated with different bevacizumab concentrations (0.25 mg/mL or 1.5 mg/mL) with or without GW4869 (10 μM or 20 μM) for 24 h and 48 h. CellTiter-Glo® luminescent cell viability assay was then performed. Results are expressed as normalised Relative Light Unit (RLU). The mean ± SEM of 4 independent experiments is shown. (*p < 0.05, ***p < 0.01; ANOVA). **c** LN18 and U87 GBM cells' invasiveness assay using a hyaluronic acid (HA) hydrogel. Cells were incubated within a HA hydrogel for 7 days. Cells were treated with bevacizumab (0.25 mg/mL) with or without GW4869 (20 μM). 5 pictures (capture) per gel were taken following the treatments. Colony counting followed by CellTiter-Glo® Luminescent Cell Viability Assay were then performed. Results are expressed as normalised number of colonies / capture and normalised RLU, respectively. The mean ± SEM of 3 independent experiments is shown (*p < 0.05; ANOVA)

The effects of EVs depletion on the viability of GBM cells in combination with bevacizumab treatment have been investigated, using GW4869, a sphyngomyelinase inhibitor shown to decrease EVs biogenesis. GW4869 inhibited EVs production (Fig. 3a), while, combination of bevacizumab with GW4869 significantly affected (~ 30%) the U87 cells’ viability after 48 h (Fig. 3b), suggesting that inhibition of EVs production could contribute to the cytotoxicity of bevacizumab in vitro. Finally, U87 invasiveness was slightly increased (~ 28%) following bevacizumab treatment [1], as assessed by their ability to form colonies in a tri-dimensional HA hydrogel (Fig. 3c), while combination with GW4869 reduced this effect. Thus, the direct anti-tumour effect of bevacizumab might be enhanced via alteration of EVs biogenesis in GBM cells. As we did not observe similar effects on LN18 invasiveness, specific molecular pathways might be implicated in the response to both bevacizumab and GW4869, as observed before [1]. It is still unclear if the additive effect on U87 cells is only due to the specific inhibition of the antibody shedding mechanisms. It might as well be a combinational effect of VEGF-A neutralization by bevacizumab along with blocking of pro-tumoral EVs (Additional file 6: Figure S4).

To what extent such mechanisms can affect bevacizumab efficacy in vivo is yet to be deciphered. Still, similar mechanisms have been described in different cancer cell types treated with antibodies. Hence, such shedding of IgG1 antibodies into GBM cells-derived EVs could be involved in GBM cells’ escape from bevacizumab. As EVs can be vividly uptaken by other cells, the destination of such bevacizumab-coated EVs is another outstanding question that require further in vivo studies.

## Conclusions

Bevacizumab has a positive effect on GBM patients’ quality of life and survival, mostly through its anti-inflammatory effects. Consequently, there is an urgent need for the right therapeutic strategy that will increase its efficacy on tumour growth, thus avoiding recurrence and relapse. Here, we report that the paradoxical pro-invasive effect of bevacizumab on GBM cells might be due to alterations in the tumour cells-derived EVs, including shedding of the antibody and further modifications of the cargo, both possibly contributing to therapeutic resistance. Therefore, this study suggests the combination of bevacizumab with a local blocking of EVs-dependent intercellular communication as a potential new therapeutic strategy to improve GBM treatment.

## Additional files

Additional file 1: Materials and methods. (DOCX 22 kb)

Additional file 2: Supplementary **Figure 1**. Direct effect of bevacizumab on LN18 and U87 GBM cells. (ZIP 375 kb)

Additional file 3: Supplementary **Figure 2**. MS protein hits identified in LN18 and U87 GBM cells-derived EVs following 24h treatment with 0.25 mg/mL IgG1/bevacizumab (FunRich analysis). (ZIP 842 kb)

Additional file 4: Supplementary **Table 1**. Mass spectrometry analysis of EVs content following treatment with IgG1/bevacizumab. **Table 2**. Mass spectrometry analysis of EVs content following treatment with IgG1/bevacizumab. (ZIP 504 kb)

Additional file 5: Supplementary **Figure 3**. Bevacizumab is detectable in GBM cells and on GBM cells-derived EVs following treatment. (ZIP 1930 kb)

Additional file 6: Supplementary **Figure 4**. Shedding of bevacizumab in tumour cells-derived extracellular vesicles as a new therapeutic resistance mechanism in glioblastoma. (ZIP 94 kb)

## Abbreviations

AAT: Anti-angiogenic therapies
CM: Conditioned medium
DMEM: Dulbecco’s Modified Eagle Medium
DTT: Dithiothreitol
EGF-R: Epidermal growth factor-receptor
ECM: Extracellular matrix
EVs: Extracellular vesicles
FBS: Fetal bovine serum
GBM: Glioblastoma
HA: Hyaluronic acid
MS: Mass spectrometry
MEM: Minimal essential medium
NTA: Nanoparticles tracking analysis
PSG: Penicillin, streptomycin, glutamine
TCGA: The cancer genome atlas
TFA: Trifluoroacetic acid
TEM: Transmission electron microscopy
VEGF-A: Vascular endothelial growth factor-A
