# Supplementary material for: Shedding of bevacizumab in tumour cells-derived extracellular vesicles as a new therapeutic escape mechanism in glioblastoma
Source: Mol Cancer. 2018 Aug 31;17:132. doi: 10.1186/s12943-018-0878-x (PMC6117885; doi:10.1186/s12943-018-0878-x)
Supplement: Supplementary file 1 — Materials and methods. (DOCX 22 kb) [file 12943_2018_878_MOESM1_ESM.docx]

**Materials and methods**

**Cells and reagents**

U87 GBM cells were maintained in Minimal Essential Medium (MEM, Sigma-Aldrich) supplemented with 100 Units/ml penicillin, 100 µg/ml streptomycin, 2 mM L-glutamine (PSG, Sigma-Aldrich) and 10% heat inactivated fetal bovine serum (FBS, First Link) at 37°C in a humidified atmosphere at 5% CO2. LN18 and LN229 GBM cells were maintained in Dulbecco’s Modified Eagle Medium (DMEM, Sigma-Aldrich) supplemented with 100 Units/ml penicillin, 100 µg/ml streptomycin, 2 mM L-glutamine and 10% heat inactivated FBS at 37°C in a humidified atmosphere at 5% CO2. Medium were changed twice a week and cells detached at confluence using trypsin/EDTA. IgG from human serum (Sigma-Aldrich) was used as an IgG1 antibody control for experiments testing bevacizumab (Brighton and Sussex University Hospitals). IgG and bevacizumab were used at 0.25mg/mL (clinically relevant concentration) or 1.5mg/mL. GW4869 (Sigma-Aldrich) was re-suspended in DMSO at 5mM and used at 10μM and 20μM.

**Cell viability assay**

Cells were plated at 5,000 cells/well in a 96 well plate in 10% FCS MEM for 24h. Then cells were washed with sterile PBS (Sigma) and treated with bevacizumab or GW4869 at indicated concentrations for 24h, 48h or 72h in 0.5% FCS or serum free medium. At the end of this incubation, 100µL of Cell-Titer-Glo® Reagent (CellTier-Glo® Luminescent Cell Viability Assay, Promega) was added to each well. Plate was agitated on a plate mixer for 2 minutes and left for 10 minutes at room temperature before luminescence was recorded.

**Cell invasiveness assay in hyaluronic acid hydrogels**

Cells were incubated with HA hydrogels for 7 days according to the manufacturer’s instructions (Biomymesis, Celenys). Colony counting was then performed on 5 pictures randomly taken from each gel using an EVOS FLC imaging system (Life Technologies) at x10 magnification. Cell viability was assessed using the CellTier-Glo® Luminescent Cell Viability Assay. To do so, 100µL of Cell-Titer-Glo® Reagent (CellTier-Glo® Luminescent Cell Viability Assay, Promega) was added to each well. Plate was agitated on a plate mixer for 2 minutes and left for 10 minutes at room temperature before luminescence was recorded.

**BrdU cell proliferation assay**

Cells were plated at 5,000 cells/well in a 96 well plate in 10% FCS MEM for 24h. Then cells were washed with sterile PBS (Sigma) and treated with bevacizumab at indicated concentrations for 24h, 48h or 72h in 0.5% FCS. At the end of this incubation, 10µL of 10X BrdU solution (BrdU Cell Proliferation Assay Kit, Cell Signaling) was added to each well and incubated for 4 hours. BrdU assay/absorbance detection (450nm) was performed as described by the manufacturer.

**Extracellular vesicles isolation**

Cells were seeded in 175cm^2^ flasks and grown in 10% FCS MEM until they reach confluence. Then, cells were washed with sterile PBS and 20mL of serum free medium was added to each flask for 24h. Treatment with bevacizumab was performed as indicated. CM was collected following this first 24h incubation and kept at -20°C for further application. Cells were then washed two times with sterile PBS and incubate an extra 24h in serum free medium before CM was collected a second time and kept at -20°C for further application. EVs were isolated using an ultracentrifugation-based protocol. Every isolation process was performed at 4°C. EVs pellets were re-suspended in 100µL filtered sterile PBS.

**Nanoparticles tracking analysis**

In order to count and determine the size of the isolated EVs, 50µL of the EVs suspension were diluted in 1mL of filtered sterile PBS and analysed using a Nanosight© NS300 (Malvern Instruments). Between 20 and 70 particles per frame were visible on the monitor. Each diluted sample was infused and 5 captures of 60s were recorded. Particles concentration per mL and their diameters could be determined using the Nanosight© NTA 3.2 software. Graphs show an average of the 5 measurements.

**Transmission Electron Microscopy**

Transmission electron microscopy (TEM) has been performed on EVs preparation in order to visualize and assess/confirm the size range of the vesicles. Immunogold labelling was performed on EVs derived from control and bevacizumab-treated GBM cells. To do so, a polyclonal Goat anti-Human IgG Fc/HRP conjugated antibody (Thermofisher) was added to the EVs preparation and left rotating overnight at 4°C. A gold-bound anti-goat secondary antibody was then added. Labelled EVs were then mounted on grids before visualization. Samples were visualized using a JEOL JEM1400-Plus (120kV, LaB6) microscope equipped with a Gatan OneView 4K camera at x20k magnification. 10-15 pictures per grid were taken.

**Western blotting**

Protein lysates were extracted using RIPA buffer (Sigma) including fresh protease and phosphatase inhibitors and standard western blotting protocol was performed. Primary antibodies used: Anti-β-Actin (Abcam), anti-Annexin A2 (Genscript), anti-CD-9 (Cell Signaling or System Biosciences), anti-EGF-R (Cell Signaling), anti-HSP-70 (System Bisociences), anti-CD44 (Cell Signaling), anti-Fibronectin (Abcam), anti-c-Met (Cell Signaling), anti-PTEN (Cell Signaling) , anti-VEGF-A (Abcam), anti-VEGF-R1 (Cell Signaling) and anti-VEGF-R2 (Cell Signaling). Secondary antibodies used: Polyclonal Goat Anti-Rabbit/Mouse Immunoglobulins/HRP (Dako) and polyclonal Goat anti-Human IgG Fc/HRP conjugated (Thermofisher) antibodies.

**Mass spectrometry**

In order to elucidate the protein content of GBM cells-derived EVs, MS analysis was performed. To do so, 50µl of the EVs suspension was mixed with loading Laemmli buffer and load on a SDS-PAGE gel. Samples were run down half of the gel before performing Coomassie blue staining. Bands were then cut out of the gel and further processed. De-staining was performed through 3 changes/washes with 50% acetonitrile (MeCN), 25mM NH_4_HCO_3_, with 5 min shaking between each change. Reduction and alkylation were performed with respectively 10 mM dithiothreitol (DTT), in 25 mM NH_4_HCO_3_ (45min at 50°C) and 50mM chloracetamide, 25mM NH_4_HCO_3_ (45min in the dark at room temperature). Subsequently, 12.5ng/µl trypsin (in 25mM NH_4_HCO_3_) was added to the samples, followed by an overnight incubation at 37°C. Next, 0.5% trifluoroacetic acid (TFA) was added to stop the trypsin digestion. Following a quick vortex and a centrifugation at high speed, supernatants containing the peptides solution was transferred to clean tubes. MeCN was then added to gel pieces in order to completely dehydrate them and consequently get the rest of the peptides. Samples volume was reduced to 20µL using a vacuum concentrator. Samples were then processed through a LTQ-Orbitrap mass spectrometer coupled to a Dionex NCP-3200 nanoLC system. Data (peaks) were searched using Mascot Daemon (Matrix Science) against a SwissProt database (Homo-Sapiens) in order to determine protein hits. The following settings were used: trypsin as the enzyme, oxydation or hydroxylation as variable modifications of the methionine, monoisotopic mass values, unrestricted protein mass, a peptide mass tolerance of 10ppm, a fragment mass tolerance of ± 0.6Da, and 1 maximum missed cleavage. Finally, results were exported to Microsoft Office Excel (significance threshold p<0.05) and further processed. Obvious contaminants (keratins) were removed from the protein hits list. Protein hits from 4 independent experiments were obtained. Hits that appeared at least 2 times out of 4 were considered Proteomic data were further deciphered by loading the gene symbols identified from the MS data in Functional Enrichment Analysis Tool (FunRich) for gene enrichment analysis of ‘Biological pathways’, ‘Cellular Compartment’ and ‘Pairwise comparison diagram’.Venn diagrams comparing the lists of genes obtained using MS data from all 3 conditions were drawn.

**VEGF-A ELISA**

Human VEGF DUOSET ELISA (R&D System) was used to measure VEGF-A levels in culture medium according to the manufacturer’s instructions. The absorbance was read at 450 nm using a GloMax Explorer plate reader (Promega).

**Trypsin resistance assay**

Trypsin resistance assays were performed using 20uL of suspension from EVs isolation. Trypsin (2.5 mg/mL, Sigma-Aldrich) was used to remove peripherally associated proteins, either in the presence or absence of 1% Triton X-100 (Sigma-Aldrich). Samples containing only the EVs suspension diluted in filtered sterile PBS or the EVs suspension diluted in Trypsin or the EVs suspension diluted in Triton or the EVs suspension diluted in a combination of Tryspsin and Triton X-100, were incubated for 30min at 37 °C. Each solution were then denatured with Laemmli buffer and analysed by western blotting.

**Immunofluorescence staining**

Cells grown on glass coverslips were fixed in 4% paraformaldehyde for 10 min, washed in PBS and incubated with 0.3% (v/v) Triton X-100 for 10 min at RT. Following, cells were washed in PBS and blocked with 10% AB serum in PBS for 30 min. Coverslips were then incubated overnight at 4°C with primary antibodies, diluted in the same buffer. Cells then were washed and incubated with secondary antibodies at RT for 60 min. After washing, coverslips were mounted onto glass slide in Mowiol (Calbiochem) with 4',6-diamidino-2-phenylindole (DAPI) solution [10% (w/v) Moviol, 1 µg/ml DAPI]. Coverslips were examined on a Leica TCS SP5 II STED laser scanning confocal microscope (Leica Microsystems). Primary antibodies used: EEA1 (Cell Signaling), Rab5 (Cell Signaling), Fibronectin (Abcam). Secondary antibodies used: Alexa Flour®-488 secondary anti-rabbit, Alexa Flour®-555 secondary anti-mouse (Invitrogen) and Alexa Fluor® 647 conjugated Goat anti-Human IgG (H+L) secondary antibodies.

**TCGA data**

Information about the distribution of specific gene hits among the different GBM subtypes has been obtained from The Cancer Genome Atlas (TCGA) through the ‘*Expression box plot (Affymetrix HT HG U133A)*’ and ‘*Expression box plot (Affymetrix Human Exon 1.0 ST)*’ graphs on the Betastasis website ([www.betastasis.com](http://www.betastasis.com)) that organize patients’ samples according to their GBM subtypes.

**Statistics**

All the results were normalised to control and reported as mean ± standard error on the mean (SEM). Data from experiments with bevacizumab was normalised to the results with IgG1. ANOVA and t-tests were employed to determine the significance of the observed differences. Differences were considered statistically significant at p<0.05 (*p<0.05; **p<0.01; ***p<0.001; ***p<0.0001).
