# Supplementary figures and images for: Shedding of bevacizumab in tumour cells-derived extracellular vesicles as a new therapeutic escape mechanism in glioblastoma
Source: Mol Cancer. 2018 Aug 31;17:132. doi: 10.1186/s12943-018-0878-x (PMC6117885; doi:10.1186/s12943-018-0878-x)

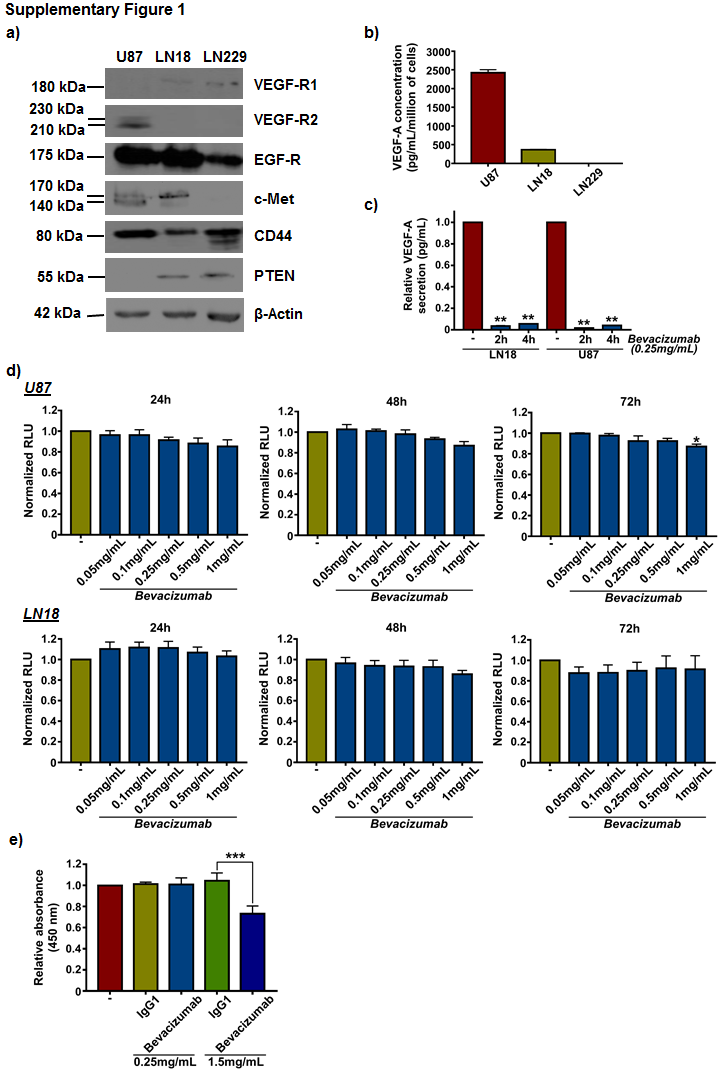

Supplement: Supplementary file 2 — Supplementary Figure 1. Direct effect of bevacizumab on LN18 and U87 GBM cells. (ZIP 375 kb) [file 12943_2018_878_MOESM2_ESM.zip › Supl. Figure 1.tif]

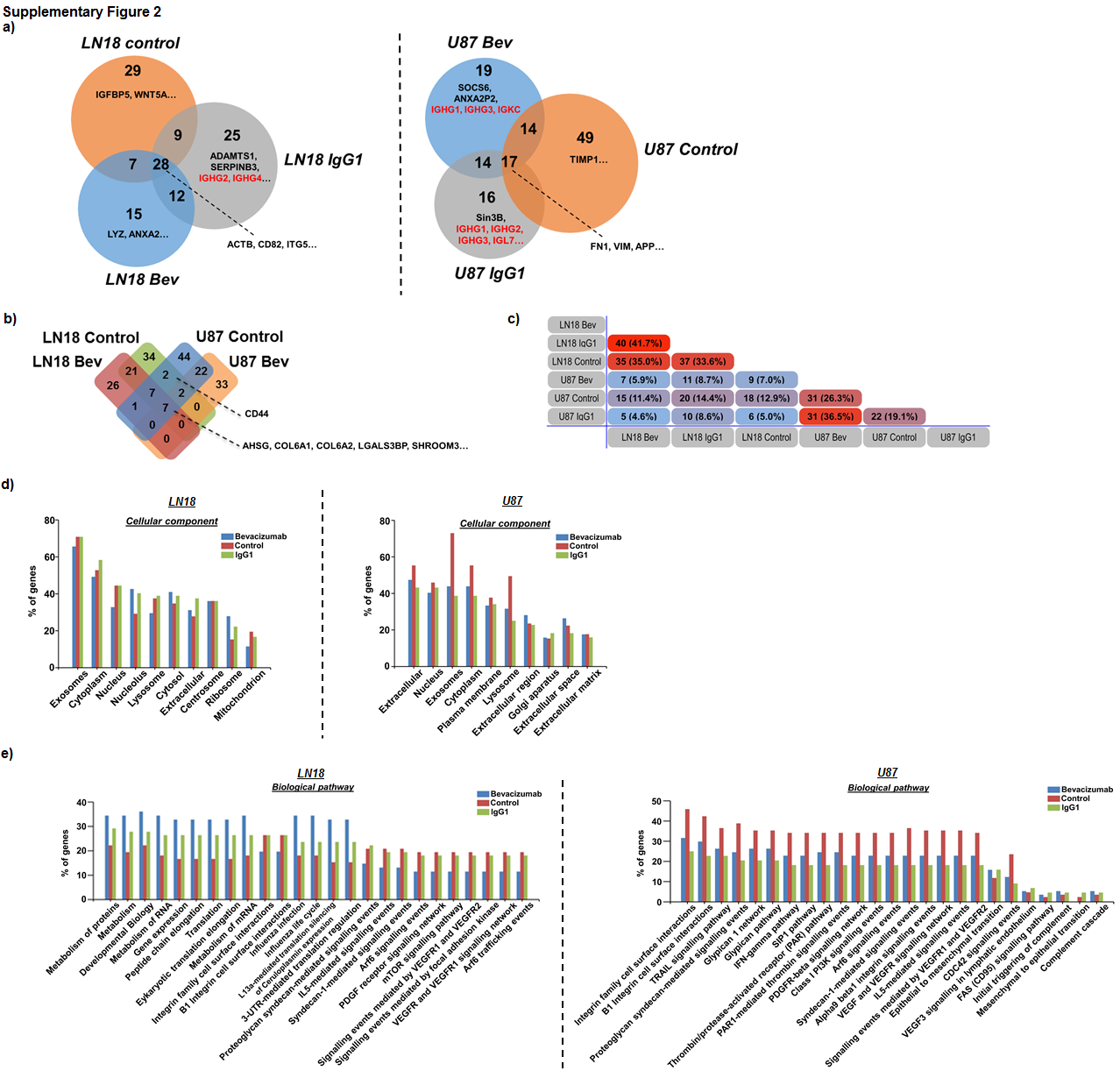

Supplement: Supplementary file 3 — Supplementary Figure 2. MS protein hits identified in LN18 and U87 GBM cells-derived EVs following 24h treatment with 0.25 mg/mL IgG1/bevacizumab (FunRich analysis). (ZIP 842 kb) [file 12943_2018_878_MOESM3_ESM.zip › Supl. Figure 2.tif]

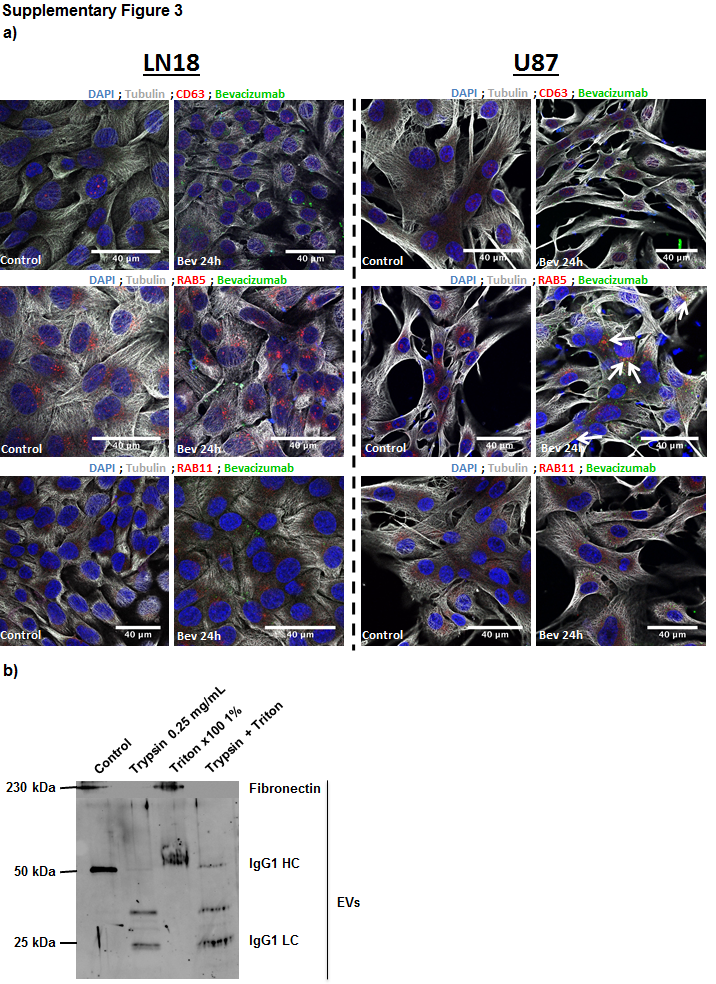

Supplement: Supplementary file 5 — Supplementary Figure 3. Bevacizumab is detectable in GBM cells and on GBM cells-derived EVs following treatment. (ZIP 1930 kb) [file 12943_2018_878_MOESM5_ESM.zip › Supl. Figure 3.tif]

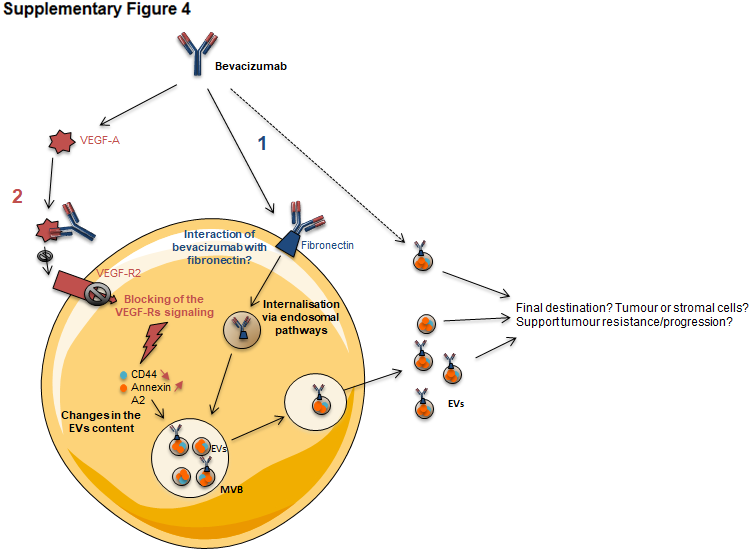

Supplement: Supplementary file 6 — Supplementary Figure 4. Shedding of bevacizumab in tumour cells-derived extracellular vesicles as a new therapeutic resistance mechanism in glioblastoma. (ZIP 94 kb) [file 12943_2018_878_MOESM6_ESM.zip › Supl. Figure 4.tif]
