# Supplementary material for: Shedding of bevacizumab in tumour cells-derived extracellular vesicles as a new therapeutic escape mechanism in glioblastoma
Source: Mol Cancer. 2018 Aug 31;17:132. doi: 10.1186/s12943-018-0878-x (PMC6117885; doi:10.1186/s12943-018-0878-x)
Supplement: Supplementary file 4 — Supplementary Table 1. Mass spectrometry analysis of EVs content following treatment with IgG1/bevacizumab. Table 2. Mass spectrometry analysis of EVs content following treatment with IgG1/bevacizumab. (ZIP 504 kb) [file 12943_2018_878_MOESM4_ESM.zip › Supl. Table 1 and Table 2.pdf]

Supplementary Table 1

Control		IgG1		Bevacizumab	
MS major hits					
Gene symbol	Protein accession number	Gene symbol	Protein accession number	Gene symbol	Protein accession number
FN1	P02751	FN1	P02751	FN1	P02751
COL6A3	P12111	LGALS3BP	Q08380	LGALS3BP	Q08380
LGALS3BP	Q08380	IGHG1	P01857	IGHG1	P01857
COL6A1	P12109	IGHG3	P01860	IGHG3	P01860
MMP2	P08253	IGHG2	P01859	A2M	P01023
HSPA5	P11021	IGLC2	P0DOY2	COL1A1	P02452
LAMC1	P11047	IGLC1	P0CG04	DCD	P81605
COL6A2	P12110	COL1A1	P02452	COL6A3	P12111
TGFBI	Q15582	IGLC7	A0M8Q6	C1S	P09871
PTGFRN	Q9P2B2	A2M	P01023	COL1A2	P08123
A2M	P01023	C1S	P09871	SHROOM3	Q8TF72
ALB	P02768	LTF	P02788	BRDT	Q58F21
C1S	P09871	DST	Q03001	OTUD6B	Q8N6M0
PLOD1	Q02809	COL1A2	P08123	COL6A2	P12110
APP	P05067	DCD	P81605	SIN3B	O75182
ECM1	Q16610	C1R	P00736	CPAMD8	Q8IZJ3
NID2	Q14112	ZC3H11A	O75152	COL6A1	P12109
HSPA2	P54652	DBF4B	Q8NFT6	IGKC	P01834
HSPA8	P11142	PIN4	Q9Y237	ASAP2	O43150
HSPA1L	P34931	ATP2B3	Q16720	GOLGA3	Q08378
ITGA3	P26006	SIN3B	O75182	ZC3H11A	O75152
C1R	P00736	COL6A1	P12109	CDY2A	Q9Y6F7
HSPA1A	P0DMV8	ASAP2	O43150	LTF	P02788
HSPA6	P17066	PPFIBP2	Q8ND30	C1R	P00736
PLOD3	O60568	ATP11AUN	Q6ZP68	SELENBP1	Q13228
LAMB1	P07942	AHSG	P02765	PDE8B	O95263
EDIL3	O43854	UTY	O14607	MMP2	P08253
C1RL	Q9NZP8	IGKC	P01834	AHSG	P02765
COL1A1	P02452	NEIL1	Q96FI4	SOCS6	O14544
PLTP	P55058	AHNAK	Q09666	TGFBI	Q15582
TNC	P24821	BARD1	Q99728	PACS1	Q6VY07
CD63	P08962	COL6A2	P12110	INADL	Q8NI35
DST	Q03001	HRNR	Q86YZ3	MKL2	Q9ULH7
NRP1	O14786	RPH3AL	Q9UNE2	HADH	Q16836
UTY	O14607	NT5DC2	Q9H857	IGHG4	P01861
CPAMD8	Q8IZJ3	THNSL2	Q86YJ6	PCOLCE	Q15113
SHROOM3	Q8TF72	CLIP4	Q8N3C7	NEFH	P12036
DCD	P81605	APP	P05067	VIM	P08670
NT5E	P21589	LAMC1	P11047	MYBL2	P10244
PRKCSH	P14314	IGHG4	P01861	RPS6KA6	Q9UK32
VIM	P08670	ECM1	Q16610	PSAP	P07602
ACTB	P60709	SDC4	P31431	SERPINC1	P01008
ITGB1	P05556	NEFH	P12036	F2	P00734
HRNR	Q86YZ3	SRGN	P10124	TF	P02787
RPS27A	P62979	INADL	Q8NI35	ITIH2	P19823
CSE1L	P55060	C17orf53	Q8N3J3	NT5E	P21589
CDY2A	Q9Y6F7	SHROOM3	Q8TF72	KIF21B	O75037
THBS2	P35442	CDY2A	Q9Y6F7	LAMC1	P11047
LTF	P02788	MKL2	Q9ULH7	HSPA5	P11021
CHI3L1	P36222	HADH	Q16836	ACTN4	O43707
PCOLCE	Q15113	TAF1B	Q53T94	C3	P01024
TIMP1	P01033	VIM	P08670	IGHG2	P01859
CLU	P10909			C1RL	Q9NZP8
CD82	P27701			APP	P05067
POTEE	Q6S8J3			HSPA6	P17066
ACTBL2	Q562R1			ANK2	Q01484
GAPDH	P04406			TAF1B	Q53T94
HTRA1	Q92743			IGLC2	P0DOY2
PPIB	P23284			GAPDH	P04406
PSMB6	P28072			CHI3L1	P36222
YWHAG	P61981			ANXA2P2	A6NMY6
YWHAZ	P63104			NRAP	Q86VF7
YWHAQ	P27348			SRRM1	Q8IYB3
YWHAB	P31946			USP17L24	Q0WX57
YWHAE	P62258				
SFN	P31947				
YWHAH	Q04917				
SERPINE1	P05121				
COL1A2	P08123				
EEF1G	P26641				
TUBB	P07437				
SDCBP	O00560				
ENO1	P06733				
CD44	P16070				
HBA1	P69905				
HBG2	P69892				
MYBL2	P10244				
PRDX1	Q06830				
CLEC11A	Q9Y240				
PDIA3	P30101				
TF	P02787				
ZBTB49	Q6ZSB9				
AHSG	P02765				
SDC4	P31431				
C3	P01024				

Supplementary Table 2

Control		IgG1		Bevacizumab	
MS major hits					
Gene symbol	Protein accession number	Gene symbol	Protein accession number	Gene symbol	Protein accession number
FN1	P02751	FN1	P02751	FN1	P02751
COL6A3	P12111	LGALS3BP	Q08380	LGALS3BP	Q08380
LGALS3BP	Q08380	IGHG1	P01857	IGHG1	P01857
COL6A1	P12109	IGHG3	P01860	IGHG3	P01860
MMP2	P08253	IGHG2	P01859	A2M	P01023
HSPA5	P11021	IGLC2	P0DOY2	COL1A1	P02452
LAMC1	P11047	IGLC1	P0CG04	DCD	P81605
COL6A2	P12110	COL1A1	P02452	COL6A3	P12111
TGFBI	Q15582	IGLC7	A0M8Q6	C1S	P09871
PTGFRN	Q9P2B2	A2M	P01023	COL1A2	P08123
A2M	P01023	C1S	P09871	SHROOM3	Q8TF72
ALB	P02768	LTF	P02788	BRDT	Q58F21
C1S	P09871	DST	Q03001	OTUD6B	Q8N6M0
PLOD1	Q02809	COL1A2	P08123	COL6A2	P12110
APP	P05067	DCD	P81605	SIN3B	O75182
ECM1	Q16610	C1R	P00736	CPAMD8	Q8IZJ3
NID2	Q14112	ZC3H11A	O75152	COL6A1	P12109
HSPA2	P54652	DBF4B	Q8NFT6	IGKC	P01834
HSPA8	P11142	PIN4	Q9Y237	ASAP2	O43150
HSPA1L	P34931	ATP2B3	Q16720	GOLGA3	Q08378
ITGA3	P26006	SIN3B	O75182	ZC3H11A	O75152
C1R	P00736	COL6A1	P12109	CDY2A	Q9Y6F7
HSPA1A	P0DMV8	ASAP2	O43150	LTF	P02788
HSPA6	P17066	PPFIBP2	Q8ND30	C1R	P00736
PLOD3	O60568	ATP11AUN	Q6ZP68	SELENBP1	Q13228
LAMB1	P07942	AHSG	P02765	PDE8B	O95263
EDIL3	O43854	UTY	O14607	MMP2	P08253
C1RL	Q9NZP8	IGKC	P01834	AHSG	P02765
COL1A1	P02452	NEIL1	Q96FI4	SOCS6	O14544
PLTP	P55058	AHNAK	Q09666	TGFBI	Q15582
TNC	P24821	BARD1	Q99728	PACS1	Q6VY07
CD63	P08962	COL6A2	P12110	INADL	Q8NI35
DST	Q03001	HRNR	Q86YZ3	MKL2	Q9ULH7
NRP1	O14786	RPH3AL	Q9UNE2	HADH	Q16836
UTY	O14607	NT5DC2	Q9H857	IGHG4	P01861
CPAMD8	Q8IZJ3	THNSL2	Q86YJ6	PCOLCE	Q15113
SHROOM3	Q8TF72	CLIP4	Q8N3C7	NEFH	P12036
DCD	P81605	APP	P05067	VIM	P08670
NT5E	P21589	LAMC1	P11047	MYBL2	P10244
PRKCSH	P14314	IGHG4	P01861	RPS6KA6	Q9UK32
VIM	P08670	ECM1	Q16610	PSAP	P07602
ACTB	P60709	SDC4	P31431	SERPINC1	P01008
ITGB1	P05556	NEFH	P12036	F2	P00734
HRNR	Q86YZ3	SRGN	P10124	TF	P02787
RPS27A	P62979	INADL	Q8NI35	ITIH2	P19823
CSE1L	P55060	C17orf53	Q8N3J3	NT5E	P21589
CDY2A	Q9Y6F7	SHROOM3	Q8TF72	KIF21B	O75037
THBS2	P35442	CDY2A	Q9Y6F7	LAMC1	P11047
LTF	P02788	MKL2	Q9ULH7	HSPA5	P11021
CHI3L1	P36222	HADH	Q16836	ACTN4	O43707
PCOLCE	Q15113	TAF1B	Q53T94	C3	P01024
TIMP1	P01033	VIM	P08670	IGHG2	P01859
CLU	P10909			C1RL	Q9NZP8
CD82	P27701			APP	P05067
POTEE	Q6S8J3			HSPA6	P17066
ACTBL2	Q562R1			ANK2	Q01484
GAPDH	P04406			TAF1B	Q53T94
HTRA1	Q92743			IGLC2	P0DOY2
PPIB	P23284			GAPDH	P04406
PSMB8	P28072			CHI3L1	P36222
YWHAG	P61981			ANXA2P2	A6NMY6
YWHAZ	P63104			NRAP	Q86VF7
YWHAQ	P27348			SRRM1	Q8IYB3
YWHAB	P31946			USP17L24	Q0WX57
YWHAE	P62258				
SFN	P31947				
YWHAH	Q04917				
SERPINE1	P05121				
COL1A2	P08123				
EEF1G	P26641				
TUBB	P07437				
SDCBP	O00560				
ENO1	P06733				
CD44	P16070				
HBA1	P69905				
HBG2	P69892				
MYBL2	P10244				
PRDX1	Q06830				
CLEC11A	Q9Y240				
PDIA3	P30101				
TF	P02787				
ZBTB49	Q6ZSB9				
AHSG	P02765				
SDC4	P31431				
C3	P01024				
