# Supplementary material for: Shedding of bevacizumab in tumour cells-derived extracellular vesicles as a new therapeutic escape mechanism in glioblastoma
Source: Mol Cancer. 2018 Aug 31;17:132. doi: 10.1186/s12943-018-0878-x (PMC6117885; doi:10.1186/s12943-018-0878-x)
Supplement: Supplementary file 2 — Supplementary Figure 1. Direct effect of bevacizumab on LN18 and U87 GBM cells. (ZIP 375 kb) [file 12943_2018_878_MOESM2_ESM.zip › Supplementary Figure 1.doc]

**Supplementary Figure 1: Direct effect of bevacizumab on LN18 and U87 GBM cells. a)** Western blotting analysis of GBM aggressiveness markers expression in three different GBM cell lines. β-actin was used as an internal control. Results from a representative experiment is shown. **b)** Analysis of VEGF-A secretion by three different GBM cell lines. Secreted VEGF-A levels were measured by ELISA assay in conditioned medium from U87, LN18 and LN229 GBM cells grown in serum-free medium for 24h. **c)** LN18 and U87 GBM cells can produce VEGF-A that is quickly neutralized by bevacizumab. Secreted VEGF-A levels were measured by ELISA assay in conditioned medium from U87 GBM cells. Human IgG was used as a control instead of bevacizumab. The mean ± SEM of 3 independent experiments is shown (**p<0.01; two-tailed paired t-test). **d)** LN18 and U87 GBM cells viability assay in response to bevacizumab. Cells were seeded in a 96w plate and allowed to grow for 24h. Cells were then treated with different bevacizumab concentrations (0.05 mg/mL, 0.1 mg/mL, 0.25 mg/mL, 0.5 mg/mL, 1 mg/mL) for 24h, 48h or 72h. CellTiter-Glor® Luminescent Cell Viability Assay was then performed. Results are expressed as Relative Light Unit (RLU). The mean ± SEM of 4 independent experiments is shown (*p<0.05; ANOVA). **e)** U87 cells proliferation in response to bevacizumab. Cells were seeded in a 96w plate and allowed to grow for 24h. Cells were then treated with different bevacizumab concentrations (0.25 mg/mL, 1.5 mg/mL) for 24h. Absorbance was read at 450nm. The mean ± SEM of 3 independent experiments is shown (***p<0.01; two-tailed paired t-test).
