# Supplementary material for: Shedding of bevacizumab in tumour cells-derived extracellular vesicles as a new therapeutic escape mechanism in glioblastoma
Source: Mol Cancer. 2018 Aug 31;17:132. doi: 10.1186/s12943-018-0878-x (PMC6117885; doi:10.1186/s12943-018-0878-x)
Supplement: Supplementary file 3 — Supplementary Figure 2. MS protein hits identified in LN18 and U87 GBM cells-derived EVs following 24h treatment with 0.25 mg/mL IgG1/bevacizumab (FunRich analysis). (ZIP 842 kb) [file 12943_2018_878_MOESM3_ESM.zip › Supplementary Figure 2.doc]

**Supplementary Figure 2: MS protein hits identified in LN18 and U87 GBM cells-derived EVs following 24h treatment with 0.25 mg/mL IgG1/bevacizumab (FunRich analysis). a)** Venn diagrams based on the identified MS hits in LN18 and U87 GBM cells-derived EVs. Gene symbols for further studied specific MS hits are shown. **b)** Venn diagram identifying the common hits in EVs derived from control GBM cells versus EVs derived from bevacizumab-treated GBM cells. **c)** Venn diagram showing the percentages of common hits between the different conditions in both LN18 and U87 GBM cells-derived EVs. **d)** Gene enrichment analysis for 10 cellular components was performed based on the MS hits identified in EVs derived from LN18 and U87 GBM cells. **e)** Gene enrichment analysis for 25 biological pathways was performed based on the MS hits identified in EVs derived from LN18 and U87 GBM cells.
