# Supplementary material for: Shedding of bevacizumab in tumour cells-derived extracellular vesicles as a new therapeutic escape mechanism in glioblastoma
Source: Mol Cancer. 2018 Aug 31;17:132. doi: 10.1186/s12943-018-0878-x (PMC6117885; doi:10.1186/s12943-018-0878-x)
Supplement: Supplementary file 4 — Supplementary Table 1. Mass spectrometry analysis of EVs content following treatment with IgG1/bevacizumab. Table 2. Mass spectrometry analysis of EVs content following treatment with IgG1/bevacizumab. (ZIP 504 kb) [file 12943_2018_878_MOESM4_ESM.zip › Supplementary Table 1 and Supplementary Table 2.doc]

**Supplementary Table 1: Mass spectrometry analysis of EVs content following treatment with IgG1/bevacizumab.** Identification of MS major hits using Mascot Daemon software. LN18 GBM cells were treated for 24h with 0.25 mg/ml IgG1 or bevacizumab. Cells were washed twice with sterile PBS and incubate for additional 24h in serum free condition before CM was collected. Then EVs were isolated from CM. EVs suspension in sterile PBS was then directly run in a SDS-PAGE gel with loading buffer. Following a Coomassie staining, bands were cut out of the gel, trypsin-digested and inject in a LTQ-Orbitrap mass spectrometer coupled to a Dionex NCP-3200 nanoLC system. Data was then processed through the Mascot Daemon software (significance threshold p<0.05). Analysis was repeated 4 times. Hits that appeared at least 2 times were considered. Gene symbols and protein accession numbers are shown.

**Supplementary Table 2: Mass spectrometry analysis of EVs content following treatment with IgG1/bevacizumab.** Identification of MS major hits using Mascot Daemon software. U87 GBM cells were treated for 24h with 0.25 mg/ml IgG1 or bevacizumab. Cells were washed twice with sterile PBS and incubate for additional 24h in serum free condition before CM was collected. Then EVs were isolated from CM. EVs suspension in sterile PBS was then directly run in a SDS-PAGE gel with loading buffer. Following a Coomassie staining, bands were cut out of the gel, trypsin-digested and inject in a LTQ-Orbitrap mass spectrometer coupled to a Dionex NCP-3200 nanoLC system. Data was then processed through the Mascot Daemon software (significance threshold p<0.05). Analysis was repeated 4 times. Hits that appeared at least 2 times were considered. Gene symbols and protein accession numbers are shown.
