# Supplementary material for: Shedding of bevacizumab in tumour cells-derived extracellular vesicles as a new therapeutic escape mechanism in glioblastoma
Source: Mol Cancer. 2018 Aug 31;17:132. doi: 10.1186/s12943-018-0878-x (PMC6117885; doi:10.1186/s12943-018-0878-x)
Supplement: Supplementary file 5 — Supplementary Figure 3. Bevacizumab is detectable in GBM cells and on GBM cells-derived EVs following treatment. (ZIP 1930 kb) [file 12943_2018_878_MOESM5_ESM.zip › Supplementary Figure 3.doc]

**Supplementary Figure 3: Bevacizumab is detectable in GBM cells and on GBM cells-derived EVs following treatment. a)** Immunofluorescence detection of bevacizumab, CD63, RAB5 and RAB11 in LN18 and U87 GBM cells. GBM cells were allowed to grow on cover slips and then treated with 0.25mg/mL bevacizumab for 2h and 24h. Cells were fixed with 4% PFA and then incubated with antibodies against α-tubulin, EEA1, CD63, RAB5, RAB11 and human IgG1. Pictures were taken at x120 magnification. Representative pictures are shown. **b)** IgG1/bevacizumab antibody is mainly bound to EVs surface. Western blotting detection of fibronectin and IgG1 antibody in LN18 GBM cells-derived EVs. Western blotting detection of bevacizumab in LN18 GBM cells-derived EVs. GBM cells were treated for 24h with 0.25 mg/ml bevacizumab. Then cells were washed two times with sterile PBS and incubated additional 24h in serum free condition without treatment. CM was then collected and EVs were isolated. EVs suspension was then diluted in a 2.5 mg/mL trypsin solution or 1% triton X100 or a trypsin/triton combination. Fibronectin and bevacizumab (IgG1) expression was analyzed by western blotting. A representative picture is shown.
