# Supplementary material for: Shedding of bevacizumab in tumour cells-derived extracellular vesicles as a new therapeutic escape mechanism in glioblastoma
Source: Mol Cancer. 2018 Aug 31;17:132. doi: 10.1186/s12943-018-0878-x (PMC6117885; doi:10.1186/s12943-018-0878-x)
Supplement: Supplementary file 6 — Supplementary Figure 4. Shedding of bevacizumab in tumour cells-derived extracellular vesicles as a new therapeutic resistance mechanism in glioblastoma. (ZIP 94 kb) [file 12943_2018_878_MOESM6_ESM.zip › Supplementary Figure 4.doc]

**Supplementary Figure 4: Shedding of bevacizumab in tumour cells-derived extracellular vesicles as a new therapeutic resistance mechanism in glioblastoma. 1.** Fibronectin-mediated trapping of bevacizumab at the EVs’ surface. IgG1 antibodies get trapped in the upper layers of the GBM cells, potentially through interaction with fibronectin. Hence, bubbling of EVs at the GBM cells’ surface causes shedding of IgG1 antibodies such as bevacizumab in tumour cells-derived extracellular vesicles. This mechanism might allow GBM cells to shield themselves against a direct anti-tumour effect of bevacizumab. **2.** Treatment with bevacizumab alters EVs proteomic content. Through altering the VEGF-A/VEGF-Rs signalling pathway by neutralization of VEGF-A, bevacizumab treatment might change the proteomic cargo of GBM cells-derived EVs. Expression of some factors involved in tumour progression regulation such as Annexin A2 or CD44 might change in GBM cells-derived Evs upon bevacizumab treatment. Furthermore, direct interaction of IgG1 antibodies with EVs surface cannot be ruled out. EV: Extracellular Vesicle; MVB: Multi Vesicular Body; VEGF-A: Vascular Endothelial Growth Factor-A; VEGF-Rs: Vascular Endothelial Growth Factor Receptors.
